# Does It Matter Who You Live with during COVID-19 Lockdown? Association of Living Arrangements with Psychosocial Health, Life Satisfaction, and Quality of Life: A Pilot Study

**DOI:** 10.3390/ijerph19031827

**Published:** 2022-02-05

**Authors:** Zijun Xu, Xiaoyang Yu, Dexing Zhang, Xiaoxiang Zheng, Zihuang Zhang, Rym Chung-Man Lee, Peter Man-Hin Cheung, Samuel Yeung-Shan Wong

**Affiliations:** 1 School of Public Health and Primary Care, The Chinese University of Hong Kong, Hong Kong 999077, China; zijunxu@link.cuhk.edu.hk (Z.X.); 1155151660@link.cuhk.edu.hk (X.Y.); ellenzheng@cuhk.edu.hk (X.Z.); chungmanrymlee@cuhk.edu.hk (R.C.-M.L.); pmhc@link.cuhk.edu.hk (P.M.-H.C.); yeungshanwong@cuhk.edu.hk (S.Y.-S.W.); 2 The Hospital for Sick Children, Toronto, ON M5X 1G8, Canada; zzha589@uwo.ca

**Keywords:** COVID-19 pandemic, lockdown, living arrangements, psychosocial health, life satisfaction, quality of life

## Abstract

Background: Living arrangements might greatly impact psychosocial health and quality of life, particularly during the COVID-19 lockdown. This pilot study aimed to examine the association of different common living arrangements with psychosocial health, life satisfaction, and quality of life among Chinese adults during the COVID-19 lockdown. Methods: An anonymous online survey was conducted using convenience sampling through the WeChat application in February 2020. Mental health (Patient Health Questionnaire-2, Generalized Anxiety Disorder-2, post-traumatic stress disorder symptoms, Patient Health Questionnaire-15, and meaning in life), social health (UCLA-3), quality of life (EQ5D and EQ-VAS), and life satisfaction were measured. Linear regression models were used. Result: The study included 1245 adults (mean age: 34.14 ± 10.71) in China. Compared to other living arrangements, participants who “live with partner and children” or “live with partner, children and parents” were more likely to have better outcomes of mental health, social health, quality of life, and life satisfaction. Participants who “live with parents or grandparents” or “live with partner” were more likely to have better health outcomes compared with those who “live with children” or “live alone”. Conclusion: Living with a partner, children, and/or parents could be a protective factor against poor psychosocial health during lockdown and quarantine.

## 1. Introduction

### 1.1. COVID-19 and Lockdown Situation in and Outside of China

Coronavirus disease 2019 (COVID-19) has been a global pandemic [1]. As of 13 December 2021, more than 269 million confirmed cases of COVID-19 have been reported worldwide, with an average mortality rate of nearly 2.0% (https://covid19.who.int/, accessed on 14 December 2021). In the early stages of the outbreak, Wuhan had far more infections and deaths than other cities in China, making Wuhan the first city in China to implement lockdown and quarantine. Private vehicles were banned in the downtown area. Highways were closed, so residents were not able to leave the city [2,3]. Due to the high infectivity of and human susceptibility to COVID-19, the Chinese government has adopted strict lockdown measures in many cities in China since January 2020, taking several measures to prevent the spread of the disease, including social distancing, self-isolation, and personal protection equipment [4]. Many public facilities, such as gyms, museums, movie theaters, and swimming pools, were closed. Residents were required to stay at home to reduce outdoor activities.

Lockdown can effectively reduce the contact between people to reduce the risk of COVID-19 infection [5]. Subsequently, many countries, such as the UK, Italy, and New Zealand, adopted similar lockdown measures for infection control. For example, in the UK, all bars, restaurants, and cafes were closed. People were asked to stay at home and avoid unnecessary social contact and travel [6].

### 1.2. Drastic Life Changes during Lockdown Make People’s Mental Health Worse

However, long-term lockdown may negatively impact people’s mental health [7]. Depression, anxiety, and insomnia were prevalent [8]. Drastic life changes, as in the case when people need to stay together with their parents, partner, and/or children for 24 h each day, might have an impact on their psychological health and quality of life. News reports showed that family disputes frequently occurred when family members were trapped in a fixed home setting with significant disturbances to their work, study, and family life for a long time due to lockdown [9,10].

Evidence showed that the pandemic increased the prevalence of symptoms of post-traumatic stress disorder (PTSD) during and after quarantine because of the loss of social contacts [7]. During the COVID-19 pandemic, a cohort study in the UK showed that people living alone had a higher risk of being lonely [11]. Another study of Spanish adults found that lower physical contact with relatives was associated with higher loneliness [12].

Living with different family members can also have different effects on psychosocial health status during a lockdown period. A cross-sectional study in Italy showed that family relationships could act as a buffer against stress if they were adequate and supportive or as a risk factor for depression if perceived as inadequate [13]. A Chinese study focusing on undergraduates showed that living with parents was a protective factor against anxiety [14]. In addition to studies conducted during the COVID-19 pandemic, an Australian study showed that those with one child had a higher risk of psychological distress than those with no children, and having three or more children appeared to be somewhat protective against high psychological distress [15]. Therefore, it is necessary to study the impact of different living arrangements, e.g., living alone or living with a partner, parents, and/or children, and provide possible recommendations for quarantine or lockdown measures once these circumstances are found to be impactful on psychological health when lockdown and quarantine are inevitable due to a pandemic.

There has never been an infectious disease outbreak like the COVID-19 pandemic in the past decades, and governments in most countries have adopted lockdown and quarantine and tried all possible ways to reduce human interaction [16]. Most of the previous studies on living arrangements were carried out in social conditions without an unexpected pandemic and large-scale lockdown [17,18]. To the best of our knowledge, research on the association between living arrangements during the lockdown and psychosocial health and quality of life during COVID-19 has been very limited to date. Most of the studies during COVID-19 only focused on living alone [19], had a simple classification of living arrangements [11,20,21,22], or were conducted in a specific population rather than the general population [23]. As a result, we conducted this cross-sectional study to understand whether who people lived with during lockdown during the COVID-19 pandemic had an association with psychosocial health, life satisfaction, and quality of life, thus informing lockdown measures and targeted interventions for more vulnerable populations. Although it may be difficult to infer causality due to the lack of indication of the sequence of events, cross-sectional studies can still indicate associations that may exist and are therefore useful in generating hypotheses for future research.

The objective of the current study was to examine the association of different living arrangements with psychosocial health, life satisfaction, and quality of life during the COVID-19 lockdown.

## 2. Materials and Methods

This was a pilot study and was conducted in line with the principles of the Declaration of Helsinki. Approval was obtained by the Survey and Behavioural Research Ethics (SBRE) Committee of the Chinese University of Hong Kong (reference no. SBRE-19-417. Dated: 20 February 2020, prior to conducting the present study).

### 2.1. Study Design and Target Population

A pilot online survey was conducted from 20 February to 12 March 2020, about 1.5 months after the outbreak in Wuhan, China. The target population was Chinese adults aged 18 years old or above. The online survey was built on a well-known and widely used survey platform (Wenjuanxing, website link: www.wjx.cn) in China. The survey invitation letter with the survey link was distributed by several investigators to potential participants using convenience and snowball sampling via WeChat, one of the most popular mobile applications for instant messaging services. The participants could complete the survey on their mobile phones, tablets, or computers. The participants were encouraged to further send the survey invitation letter and link to their friends and family members. All participants were voluntarily engaged in the research and could terminate the survey at any time. The survey took about 10 min to complete and was anonymous and confidential. A quality check was conducted by adding two repeated questions at the end of the survey to test if participants diligently answered the questions. The participants were required to provide the same or similar answers to the two repeated questions to be considered eligible. Furthermore, the response time for the survey was required to be at least 250 s, and one mobile or computer could only respond once. After completing the survey, all eligible participants received a lucky draw of 1–10 RMB, a report on their physical and mental health status, and information on hotlines and institutions for psychological help. The study design can also be found in our previous publications [24,25]. A total of 1742 individuals completed the questionnaire, but 268 of the participants did not pass the quality check process, and 18 were under the age of 18. Among the remaining 1456 participants who completed the survey, this study included 1245 participants who lived with a partner, children, parents, and/or grandparents or lived alone.

### 2.2. Measurements

#### 2.2.1. Living Arrangement

The participants were asked “Who do you live with at this moment?” and the answers included “partner”, “children”, “parents”, “grandparents”, “grandchildren”, “hotel or group residence”, “live alone”, and “others”. The participants were allowed to choose one or more answers.

#### 2.2.2. Mental Health

The two-item Patient Health Questionnaire (PHQ-2, Cronbach’s α = 0.086, sensitivity = 0.86, specificity = 0.86) and the two-item Generalized Anxiety Disorder Questionnaire (GAD-2, Cronbach’s α = 0.88, sensitivity = 0.94, specificity = 0.91) were used to screen depressive and anxiety symptoms and were validated in Chinese [26,27]. Higher scores indicate higher severity [28,29]. The total score of two items—recurrent dreams and avoidance of the COVID-19 epidemic—from the Clinician-Administered Post-Traumatic Stress Disorder (PTSD, sensitivity = 0.88, specificity = 0.71) Scale was used to assess PTSD symptoms due to the COVID-19 epidemic [30]. The possible score of each item ranged from 1 (absent) to 5 (severe). The Chinese-validated 15-item Patient Health Questionnaire (PHQ-15, Cronbach’s α = 0.79, sensitivity = 0.78, specificity = 0.71) was used to measure somatic symptoms [31]. A higher score indicated more somatic symptoms (range of 0–30). Meaning in life was measured by one item of personal existence on a 7-point scale from 1 (utterly meaningless and without purpose) to 7 (very purposeful and meaningful), which was extracted from the validated reliable Chinese Purpose in Life test (CPIL) [32].

#### 2.2.3. Social Health

A three-item Chinese-validated UCLA Loneliness Scale (UCLA-3, Cronbach’s α = 0.89) was used to measure loneliness (range 3–9), with higher scores representing more severe loneliness [33].

#### 2.2.4. Life Satisfaction and Quality of Life

A validated Chinese version of the European Quality of Life 5-dimension (EQ5D) Questionnaire and its visual analog scale (EQ-VAS, ranging from 0 to 100) were used to measure health-related quality of life, with higher scores signifying better quality of life [34]. Life satisfaction was assessed by one item: “Are you satisfied with your life?” on a 7-point Likert scale ranging from 1 (very dissatisfied) to 7 (very satisfied).

### 2.3. Statistical Analysis

The outcomes are presented as frequency and percentage for categorical variables or mean and standard deviation (SD) for continuous variables. A Kruskal–Wallis rank test was used to identify differences between continuous variables among those who lived with different family members. The outcomes were also included in univariable and multiple linear regression models before and after adjusting for age, gender, education, and job, respectively. The estimates of the strengths of the association between living arrangements and variables of interest were demonstrated by adjusted coefficients and their corresponding 95% confidence interval (CI). All data were analyzed using Stata version 16.0, and the significance level was set at *p* < 0.05 (2-tailed).

## 3. Results

### 3.1. Demographic Characteristics and Distribution of Outcome Scores

The study included 1245 participants with a mean age of 34.14 ± 10.71 years (range: 18–77 years). Of all participants, 57.6% were female, 36.9% were single, 61.0% were married, 69.6% were employed, and 86.8% had an education level of college and above (Table 1).

The results of the overall health profiles of the participants as well as participants with different living arrangements are shown in Table 2. In terms of mental health, the scores of PHQ-2, GAD-2, PTSD symptoms, PHQ-15, and meaning in life were 1.02 ± 1.30, 0.83 ± 1.18, 2.55 ± 0.98, 3.87 ± 4.00, and 5.83 ± 1.32, respectively. The included participants had a UCLA-3 score of 3.83 ± 1.26. Regarding life satisfaction and quality of life, the scores of EQ5D, EQ-VAS, and life satisfaction were 0.91 ± 0.14, 83.14 ± 18.44, and 4.71 ± 1.58, respectively.

### 3.2. Association with Different Living Arrangements

According to the Kruskal–Wallis rank test results in Table 2, living arrangements were significantly associated with the positive rate of PHQ-2, the positive rate of GAD-2, PHQ-15 score, meaning in life, social health (loneliness), life satisfaction, and quality of life (both index and VAS scores), i.e., all characteristics except for PTSD symptoms.

Table 3 and Table 4 provide the results of the univariable and multiple linear regression models before and after adjustment, respectively, for predicting mental health, social health, life satisfaction, and quality of life. When compared with “living alone” in the multiple analysis (Table 4), participants who “live with partner” had a significantly better score in UCLA-3; those who “live with parents or grandparents” had better scores on PHQ-2, UCLA-3, and EQ-VAS; those who “live with partner and children” had better scores in PHQ-2, GAD-2, meaning in life, UCLA-3, EQ-VAS, and life satisfaction; those who “live with partner, children and parents” had better scores on PHQ-2, GAD-2, meaning in life, UCLA-3, EQ5D, and life satisfaction (*p* < 0.05). There was no difference between participants who “live alone” and those who “live with children” (*p* > 0.05).

In the multiple regression after adjustment (Table 4), compared with participants who “live with children”, those who “live with partner” had significantly better outcomes in GAD-2 and UCLA-3; those who “live with parents or grandparents” had a better score in UCLA-3; both participants who “live with partner and children” and “live with partner, children and parents” had better scores on PHQ-2, GAD-2, PHQ-15, UCLA-3, and life satisfaction (*p* < 0.05).

When compared with participants who “live with partner” (Table 4), both participants who “live with partner and children” and “live with partner, children and parents” had better scores on PHQ-15 and meaning in life, while those who “live with partner and children” also performed better in PHQ-2 and UCLA-3 (*p* < 0.05). However, there was no difference between participants who “live with partner” and those who “live with parents or grandparents” (*p* > 0.05).

When compared with “living with parents or grandparents” (Table 4), participants who “live with partner and children” had better scores on meaning in life, UCLA-3, and life satisfaction; those who “live with partner, children and parents” had better scores on GAD-2, meaning in life, and life satisfaction (*p* < 0.05). However, no significant difference was found between participants who “live with partner and children” and those who “live with partner, children and parents” (*p* > 0.05).

## 4. Discussion

### 4.1. Major Findings

This pilot study aimed to provide an overview of the impact and explore which type of living arrangements might be related to better psychosocial health, life satisfaction, and quality of life among Chinese adults during the COVID-19 lockdown. A major finding of this study is that scores on PHQ-2, GAD-2, PHQ-15, UCLA-3, EQ5D, EQ-VAS, meaning in life, and life satisfaction were statistically different among various living arrangements, indicating that living with different family members was associated with the mental health, social health, life satisfaction, and quality of life of the participant. In general, participants who lived with a partner, children, and/or parents tended to have better psychosocial health, followed by those who lived with a partner or parents, while those living with children or living alone had the poorest psychosocial health.

There might be two main reasons to explain the above results. The first is the sense of purpose from family life. Some studies have confirmed that family life is an important component of thriving and well-being [35]. In particular, the pursuit of meaning in life contributes to the enhancement of life quality, life satisfaction, and mental health [36,37]. A previous study also found that family identification has a significant impact on quality of life [38]. Soares et al. pointed out that those living in large households were associated with a better quality of life than those living in small households or alone [39], with evidence supporting that living with a larger number of people during the COVID-19 outbreak was associated with better mental health and life satisfaction [40]. The second is social support from family members. Lau et al. observed that many residents in Hong Kong were not seriously affected by the SARS epidemic because of increased family support and care during that time [41]. Another study in the Netherlands during COVID-19 found that living with a spouse or partner was associated with a decreased risk of loneliness [42]. On the other hand, living alone or only with children means having less family support during the COVID-19 lockdown. They can feel a greater sense of the absence of social contacts caused by lockdown at home [21]. People who live only with children also need to take on all of the responsibility of taking care of their children without support from their partner and other family members, which might add additional mental, physical, and financial pressures. A study during the COVID-19 pandemic in the UK found that living with children, especially young children aged under five, significantly increased the individual’s mental distress compared with that before the COVID-19 epidemic [43]. The study of Coppola et al. indicated that during COVID-19, people living with children had worse mental health [22]. Another study during COVID-19 in China found that people living alone had 1.68 times the risk of depressive and anxiety symptoms than those who lived with others [44]. In addition, the results showed no significant differences during the COVID-19 lockdown among certain groups: (1) people who live with parent(s), a partner, and children vs. people who live with a partner and children, (2) people who live with parent(s) vs. people who live with a partner, and (3) people who live alone vs. people who live only with children. These findings might suggest that social support is essentially important during the lockdown, while parents and partners might have a similar role in social support. However, future studies still need to take a closer look at these possibilities.

### 4.2. Strengths and Limitations

This study examined the association of living arrangements with psychosocial health, life satisfaction, and quality of life during the COVID-19 lockdown, and therefore, it can help to provide a better understanding of the relationship between mental health and the role of living with different family members during the lockdown, as well as provide a further reference for lockdown and quarantine arrangements for residents during the COVID-19 pandemic or other crises.

However, our study had some limitations that need to be addressed. First, the data were collected by snowball sampling, which is a kind of non-probability sampling. Some specific environmental factors, which may relate to the researchers, were not taken into account in the analysis and interpretation of results. In addition, the questionnaire was generated and distributed via an online platform and WeChat mobile application. Children and adolescents under 18 and residents who do not have mobile phones were not included, so the result cannot be generalized to all residents in China. Second, the primary study is a cross-sectional study and cannot establish a causal relationship between different living arrangements and psychosocial health and quality of life during the COVID-19 lockdown. Longitudinal studies are needed for further confirmation of the causal relationship. Third, we only studied common types of living arrangements and did not study other types of living arrangements, e.g., living in a dormitory, with friends, with pets, or with both a partner and parents, as these living arrangements were not common in our study sample. Furthermore, we did not specifically look into the impact of relationships of different family members. Future studies can examine other types of living arrangements and family relationships as well.

### 4.3. Implications

Currently, the most common quarantine policy for close contacts and overseas returning travelers is that, except for young children and other people with an indication of severe medical needs, people must isolate in single rooms for medical observation. A successful quarantine can control the epidemic while minimizing its adverse psychological impacts at the same time. The quarantine and lockdown policy can be appropriately relaxed, and available resources should be properly arranged, allowing families to live together for better psychosocial health, life satisfaction, and quality of life during the period of isolation. Special attention and more support should also be provided to people who live alone or only with children under quarantine or lockdown.

## 5. Conclusions

In summary, the findings of this study demonstrate that various living arrangements were associated with differences in mental health, social health, life satisfaction, and quality of life. In general, participants who lived with a partner, children, and/or parents tended to have better psychosocial health, followed by those who lived with a partner or parents, than those living with children or living alone. Follow-up studies are necessary to confirm the causal relationship and long-term impacts. In addition, lockdown and quarantine measures should account for the living arrangement and family composition and pay special attention to those who live alone or live only with children.

## Figures and Tables

**Table 1:** Demographic characteristics of the study participants (n = 1245).

Characteristics	Category	Mean ± SD/n (%)
Age (years)		34.14 ± 10.71
Gender	Male	528 (42.4)
	Female	717 (57.6)
Marital status	Single	459 (36.9)
	Married	760 (61.0)
	Separate/Divorce/Widowed	26 (2.1)
Work	Employed	866 (69.6)
	Unemployed	99 (8.0)
	Student	262 (21.0)
	Unknown	18 (1.4)
Education level	High school and below	164 (13.2)
	College	185 (14.9)
	Bachelor	567 (45.5)
	Postgraduate and above	329 (26.4)

**Table 2:** Distribution of outcome scores among adults with different living arrangements.

Characteristics	Total	Alone	Only Children	Only Parents/ Grandparents	Only Partner			Partner and Children			Partner, Children and Parents	*p* ^a^
Mental health												
PHQ-2	1.02 ± 1.30	1.35 ± 1.47	1.31 ± 1.76	1.20 ± 1.34	0.96 ± 1.24			0.73 ± 1.17			0.84 ± 1.13	<0.001
GAD-2	0.83 ± 1.18	1.11 ± 1.32	1.16 ± 1.61	1.00 ± 1.31	0.74 ± 0.99			0.65 ± 1.04			0.61 ± 0.98	<0.001
PTSD symptoms	2.55 ± 0.98	2.62 ± 1.04	2.49 ± 1.04	2.66 ± 1.11	2.45 ± 0.86			2.52 ± 0.91			2.46 ± 0.82	0.243
PHQ-15	3.87 ± 4.00	3.95 ± 3.83	5.00 ± 5.73	4.44 ± 4.18	4.21 ± 4.02			3.22 ± 3.62			3.09 ± 3.55	<0.001
Meaning in life	5.83 ± 1.32	5.53 ± 1.37	5.93 ± 1.16	5.50 ± 1.44	5.83 ± 1.40			6.22 ± 1.10			6.11 ± 1.08	<0.001
Social Health												
UCLA-3	3.83 ± 1.26	4.34 ± 1.41	4.31 ± 1.61	3.93 ± 1.32	3.76 ± 1.16			3.56 ± 1.06			3.65 ± 1.16	<0.001
Life satisfaction and quality of life												
EQ5D	0.91 ± 0.14	0.89 ± 0.18	0.89 ± 0.13	0.91 ± 0.15	0.92 ± 0.12			0.92 ± 0.12			0.93 ± 0.14	0.046
EQ-VAS	83.14 ± 18.44	78.62 ± 22.21	80.27 ± 22.58	84.34 ± 17.96	83.55 ± 17.42			84.69 ± 16.19			81.99 ± 18.92	0.009
Life satisfaction	4.71 ± 1.58	4.44 ± 1.54	4.58 ± 1.47	4.26 ± 1.61	4.96 ± 1.58			5.08 ± 1.49			5.04 ± 1.47	<0.001
			Worse outcome								Better outcome	

^a^ Kruskal–Wallis rank test was used. Results are summarized as mean ± SD. Results highlighted in green mean better outcomes compared with the reference. Results highlighted in red mean worse outcomes compared with the reference.

**Table 3:** Association of different living arrangements with various outcomes before adjustment.

Comparisons		Mental Health					Social Health		Life Satisfaction and Quality of Life	
		PHQ-2	GAD-2	PTSD Symptoms	PHQ-15	Meaning in Life	UCLA-3	EQ5D	EQ-VAS	Life Satisfaction
Only children	vs. Alone	−0.03 (−0.47, 0.40)	0.04 (−0.35, 0.44)	−0.14 (−0.46, 0.19)	1.05 (−0.29, 2.39)	0.40 (−0.04, 0.84)	−0.03 (−0.45, 0.39)	0.00 (−0.05, 0.05)	1.64 (−4.57, 7.86)	0.13 (−0.39, 0.66)
Only partner		−0.39 (−0.68, −0.10) ** ^a^	−0.38 (−0.64, −0.11) ** ^a^	−0.18 (−0.39, 0.04)	0.27 (−0.62, 1.15)	0.29 (0.00, 0.58) * ^a^	−0.58 (−0.86, −0.30) *** ^a^	0.03 (−0.01, 0.06)	4.92 (0.81, 9.03) * ^a^	0.51 (0.17, 0.86) ** ^a^
Only parents/ grand-parents		−0.14 (−0.40, 0.11)	−0.12 (−0.35, 0.12)	0.04 (−0.15, 0.23)	0.49 (−0.29, 1.28)	−0.03 (−0.28, 0.22)	−0.41 (−0.65, −0.17) ** ^a^	0.02 (−0.01, 0.05)	5.71 (2.09, 9.34) ** ^a^	−0.19 (−0.49, 0.12)
Partner and children		−0.61 (−0.88, −0.35) *** ^a^	−0.46 (−0.70, −0.22) *** ^a^	−0.10 (−0.30, 0.10)	−0.72 (−1.54, 0.09)	0.68 (0.42, 0.95) *** ^a^	−0.78 (−1.04, −0.53) *** ^a^	0.03 (0.00, 0.06) * ^a^	6.06 (2.27, 9.85) ** ^a^	0.64 (0.32, 0.96) *** ^a^
Partner, children, and parents		−0.50 (−0.78, −0.22) *** ^a^	−0.50 (−0.76, −0.25) *** ^a^	−0.17 (−0.38, 0.04)	−0.85 (−1.71, 0.01)	0.58 (0.30, 0.86) *** ^a^	−0.69 (−0.96, −0.42) *** ^a^	0.04 (0.01, 0.07) * ^a^	3.37 (−0.62, 7.35)	0.59 (0.26, 0.93) ** ^a^
Only partner	vs. Only children	−0.35 (−0.78, 0.07)	−0.42 (−0.80, −0.04) *	−0.04 (−0.36, 0.28)	−0.79 (−2.08, 0.51)	−0.11 (−0.53, 0.31)	−0.55 (−0.96, −0.15) ** ^a^	0.03 (−0.02, 0.07)	3.28 (−2.72, 9.28)	0.38 (−0.13, 0.88)
Only parents/ grandparents		−0.11 (−0.50, 0.29)	−0.16 (−0.52, 0.20)	0.17 (−0.13, 0.47)	−0.56 (−1.78, 0.67)	−0.43 (−0.83, −0.03) *** ^b^	−0.38 (−0.77, 0.00) * ^a^	0.02 (−0.03, 0.06)	4.07 (−1.61, 9.75)	−0.32 (−0.80, 0.16)
Partner and children		−0.58 (−0.98, −0.17) ** ^a^	−0.50 (−0.87, −0.14) ** ^a^	0.03 (−0.27, 0.34)	−1.78 (−3.02, −0.53) ** ^a^	0.28 (−0.12, 0.69)	−0.76 (−1.15, −0.37) *** ^a^	0.03 (−0.01, 0.07)	4.42 (−1.37, 10.2)	0.50 (0.02, 0.99) * ^a^
Partner, children, and parents		−0.47 (−0.88, −0.05) * ^a^	−0.55 (−0.92, −0.17) ** ^a^	−0.03 (−0.34, 0.28)	−1.91 (−3.18, −0.63) ** ^a^	0.18 (−0.24, 0.59)	−0.66 (−1.06, −0.26) ** ^a^	0.04 (−0.01, 0.08)	1.72 (−4.19, 7.64)	0.46 (−0.04, 0.96)
Only parents/ grandparents	vs. Only partner	0.25 (0.02, 0.47) * ^b^	0.26 (0.05, 0.47) * ^b^	0.21 (0.04, 0.38) * ^b^	0.23 (−0.47, 0.93)	−0.32 (−0.55, −0.09) ** ^b^	0.17 (−0.05, 0.39)	−0.01 (−0.03, 0.02)	0.79 (−2.44, 4.02)	−0.70 (−0.97, −0.43) *** ^b^
Partner and children		−0.22 (−0.46, 0.02)	−0.09 (−0.30, 0.13)	0.07 (−0.11, 0.26)	−0.99 (−1.72, −0.25) ** ^a^	0.39 (0.15, 0.63) ** ^a^	−0.21 (−0.44, 0.02)	0.00 (−0.02, 0.03)	1.14 (−2.28, 4.55)	0.12 (−0.16, 0.41)
Partner, children, and parents		−0.11 (−0.37, 0.14)	−0.13 (−0.36, 0.10)	0.01 (−0.18, 0.20)	−1.12 (−1.9, −0.33) ** ^a^	0.29 (0.03, 0.54) * ^a^	−0.11 (−0.35, 0.14)	0.01 (−0.02, 0.04)	−1.56 (−5.19, 2.08)	0.08 (−0.22, 0.39)
Partner and children	vs. Only parents/ grandparents	−0.47 (−0.67, −0.27) *** ^a^	−0.34 (−0.52, −0.17) *** ^a^	−0.14 (−0.29, 0.01)	−1.22 (−1.82, −0.61) *** ^a^	0.71 (0.52, 0.91) *** ^a^	−0.37 (−0.56, −0.18) *** ^a^	0.01 (−0.01, 0.03)	0.35 (−2.47, 3.16)	0.82 (0.59, 1.06) *** ^a^
Partner, children, and parents		−0.36 (−0.58, −0.14) ** ^a^	−0.39 (−0.58, −0.19) *** ^a^	−0.20 (−0.37, −0.04) * ^a^	−1.35 (−2.01, −0.68) *** ^a^	0.61 (0.39, 0.83) *** ^a^	−0.28 (−0.48, −0.07) ** ^a^	0.02 (0.00, 0.04)	−2.35 (−5.43, 0.74)	0.78 (0.52, 1.04) *** ^a^
Partner, children, and parents	vs. Partner and children	0.11 (−0.12, 0.34)	−0.04 (−0.25, 0.16)	−0.07 (−0.24, 0.11)	−0.13 (−0.83, 0.57)	−0.10 (−0.33, 0.12)	0.10 (−0.12, 0.32)	0.01 (−0.02, 0.03)	−2.69 (−5.96, 0.58)	−0.04 (−0.32, 0.23)

* *p* < 0.05; ** *p* < 0.01; *** *p* < 0.001. ^a^ Results highlighted in green mean better outcomes compared with the reference. ^b^ Results highlighted in red mean worse outcomes compared with the reference. Results are presented as regression coefficient β (95% CI) adjusted for age, gender, education, and job.

**Table 4:** Association of different living arrangements with various outcomes after adjustment.

Comparisons		Mental Health					Social Health		Life Satisfaction and Quality of Life	
		PHQ-2	GAD-2	PTSD Symptoms	PHQ-15	Meaning in Life	UCLA-3	EQ5D	EQ-VAS	Life Satisfaction
Only children	vs. Alone	0.13 (−0.31, 0.58)	0.16 (−0.25, 0.57)	−0.02 (−0.35, 0.32)	1.02 (−0.35, 2.39)	0.29 (−0.16, 0.73)	−0.02 (−0.45, 0.41)	0.00 (−0.05, 0.04)	0.77 (−5.63, 7.18)	−0.04 (−0.57, 0.49)
Only partner		−0.20 (−0.50, 0.11)	−0.24 (−0.52, 0.04)	−0.06 (−0.29, 0.17)	0.52 (−0.42, 1.45)	0.15 (−0.16, 0.45)	−0.52 (−0.82, −0.23) *** ^a^	0.02 (−0.01, 0.06)	4.29 (−0.08, 8.67)	0.27 (−0.09, 0.64)
Only parents/grandparents		−0.27 (−0.55, 0.00) * ^a^	−0.19 (−0.44, 0.06)	0.05 (−0.15, 0.26)	0.06 (−0.78, 0.89)	0.12 (−0.15, 0.39)	−0.47 (−0.74, −0.21) *** ^a^	0.01 (−0.02, 0.04)	5.9 (1.99, 9.81) ** ^a^	0.01 (−0.32, 0.33)
Partner and children		−0.46 (−0.74, −0.18) ** ^a^	−0.35 (−0.6, −0.09) ** ^a^	0.00 (−0.21, 0.21)	−0.50 (−1.37, 0.36)	0.56 (0.28, 0.85) *** ^a^	−0.76 (−1.03, −0.49) *** ^a^	0.03 (0.00, 0.06)	5.61 (1.58, 9.64) ** ^a^	0.49 (0.16, 0.83) ** ^a^
Partner, children, and parents		−0.40 (−0.68, −0.12) ** ^a^	−0.43 (−0.69, −0.18) ** ^a^	−0.13 (−0.34, 0.08)	−0.72 (−1.59, 0.15)	0.51 (0.22, 0.79) *** ^a^	−0.68 (−0.95, −0.40) *** ^a^	0.04 (0.01, 0.07) * ^a^	3.09 (−0.98, 7.16)	0.50 (0.16, 0.84) ** ^a^
Only partner	vs. Only children	−0.33 (−0.75, 0.09)	−0.40 (−0.78, −0.02) * ^a^	−0.05 (−0.36, 0.27)	−0.50 (−1.79, 0.80)	−0.14 (−0.56, 0.29)	−0.5 0 (−0.91, −0.10) * ^a^	0.03 (−0.02, 0.07)	3.52 (−2.54, 9.58)	0.31 (−0.19, 0.81)
Only parents/grandparents		−0.41 (−0.84, 0.02)	−0.35 (−0.74, 0.04)	0.07 (−0.26, 0.39)	−0.96 (−2.28, 0.36)	−0.17 (−0.60, 0.27)	−0.45 (−0.86, −0.04) * ^a^	0.02 (−0.03, 0.06)	5.12 (−1.05, 11.3)	0.04 (−0.47, 0.56)
Partner and children		−0.59 (−0.99, −0.18) ** ^a^	−0.51 (−0.88, −0.14) ** ^a^	0.02 (−0.29, 0.32)	−1.52 (−2.77, −0.27) * ^a^	0.28 (−0.13, 0.69)	−0.74 (−1.13, −0.35) *** ^a^	0.03 (−0.01, 0.08)	4.83 (−1.01, 10.68)	0.53 (0.04, 1.01) * ^a^
Partner, children, and parents		−0.53 (−0.95, −0.12) * ^a^	−0.59 (−0.97, −0.21) ** ^a^	−0.11 (−0.43, 0.20)	−1.73 (−3.02, −0.45) ** ^a^	0.22 (−0.20, 0.64)	−0.65 (−1.06, −0.25) ** ^a^	0.04 (0.00, 0.09)	2.31 (−3.69, 8.32)	0.54 (0.04, 1.03) * ^a^
Only parents/grandparents	vs. Only partner	−0.08 (−0.36, 0.21)	0.05 (−0.21, 0.31)	0.11 (−0.10, 0.33)	−0.46 (−1.35, 0.42)	−0.03 (−0.32, 0.26)	0.05 (−0.22, 0.33)	−0.01 (−0.04, 0.02)	1.61 (−2.52, 5.73)	−0.27 (−0.61, 0.08)
Partner and children		−0.26 (−0.50, −0.02) * ^a^	−0.11 (−0.33, 0.11)	0.06 (−0.12, 0.24)	−1.02 (−1.77, −0.28) ** ^a^	0.42 (0.17, 0.66) ** ^a^	−0.24 (−0.47, −0.01) * ^a^	0.00 (−0.02, 0.03)	1.31 (−2.16, 4.79)	0.22 (−0.07, 0.50)
Partner, children, and parents		−0.20 (−0.46, 0.06)	−0.19 (−0.43, 0.05)	−0.07 (−0.26, 0.13)	−1.24 (−2.04, −0.43) ** ^a^	0.36 (0.09, 0.62) ** ^a^	−0.15 (−0.4, 0.10)	0.02 (−0.01, 0.04)	−1.2 (−4.96, 2.56)	0.23 (−0.08, 0.54)
Partner and children	vs. Only parents/ grandparents	−0.18 (−0.44, 0.08)	−0.16 (−0.39, 0.08)	−0.05 (−0.25, 0.14)	−0.56 (−1.36, 0.24)	0.44 (0.18, 0.70) ** ^a^	−0.29 (−0.54, −0.04) * ^a^	0.01 (−0.01, 0.04)	−0.29 (−4.02, 3.44)	0.48 (0.17, 0.79) ** ^a^
Partner, children, and parents		−0.13 (−0.39, 0.13)	−0.24 (−0.48, −0.01) * ^a^	−0.18 (−0.38, 0.01)	−0.77 (−1.57, 0.02)	0.38 (0.13, 0.64) ** ^a^	−0.20 (−0.45, 0.05)	0.03 (0.00, 0.05)	−2.81 (−6.53, 0.92)	0.49 (0.18, 0.80) ** ^a^
Partner, children, and parents	vs. Partner and children	0.05 (−0.18, 0.29)	−0.09 (−0.30, 0.13)	−0.13 (−0.30, 0.05)	−0.21 (−0.93, 0.51)	−0.06 (−0.29, 0.18)	0.09 (−0.14, 0.31)	0.01 (−0.01, 0.04)	−2.52 (−5.88, 0.85)	0.01 (−0.27, 0.29)

* *p* < 0.05; ** *p* < 0.01; *** *p* < 0.001. ^a^ Results highlighted in green mean better outcomes compared with the reference. Results are presented as regression coefficient β (95% CI) adjusted for age, gender, education, and job.

## Data Availability

The data presented in this study are available on request from the corresponding author. The data are not publicly available because the study is not yet finished.
